# Short-term simulation training course improves basic obstetric ultrasound operational skills of residents: A multi-center study

**DOI:** 10.1371/journal.pone.0328122

**Published:** 2025-12-26

**Authors:** Yongfeng Zhao, Ping Zhou, Xiaohong Tang, Wenhui Zhu, Wengang Liu, Jinguang Zhou, Jie Wang, Yingchun Luo, Junhui Zhang, Lan Yang, Yan Xu

**Affiliations:** 1 Department of Ultrasound, The Third Xiangya Hospital of Central South University, Changsha, China; 2 Clinical skill center, The Third Xiangya Hospital of Central South University, Changsha, China; 3 Department of Ultrasound, Hunan Provincial Maternal and Child Health Care Hospital, Changsha, China; 4 Department of Ultrasound, Nanjing Drum Tower Hospital, Nanjing, China; Guilan University of Medical Sciences, IRAN, ISLAMIC REPUBLIC OF

## Abstract

**Objectives:**

To evaluate effectiveness of a short-term simulation-based obstetric ultrasound training course.

**Methods:**

21 inexperienced trainees and 44 experienced trainees participated in a short-term course about obstetric ultrasound operational skills, which was designed into four task modules. Participants deliberately practiced on simulators for one hour at each module. Their pre-training and post-training operational skill levels were assessed using Obstetric ultrasound competency assessment tool. Likert scale was used to learn about trainees’ confidence before and after training, and their satisfaction with the simulated training course.

**Results:**

Scores of inexperienced and experienced trainees after training were significantly higher than those before training (inexperienced trainees 20.48 ± 10.64 vs 61.80 ± 15.40, P < 0.001; experienced trainees 39.83 ± 15.00 vs 76.26 ± 12.16, P < 0.001). The passing rate increased from 0% to 90.5% for inexperienced trainees, and from 31.8% to 97.7% for experienced trainees (all P < 0.001). The mastery learning rate increased from 0% to 23.8% for inexperienced trainees, and from 0% to 70.5% for experienced trainees (all P < 0.001). Trainees’ confidence in obstetric ultrasound examination also significantly improved compared to pre-training (2.66 ± 1.12 vs 3.98 ± 0.50, P < 0.001). Most trainees believed that simulation training could help them acquire standardized ultrasound planes (58/60), form organized thinking of scanning (59/60) and improve their competence in following clinical practice (54/60).

**Conclusions:**

Both inexperienced and experienced trainees can benefit from the short-term simulation training course. Trainees demonstrated strong training satisfaction with the simulation training course.

## Introduction

Ultrasound is vital for prenatal diagnosis, yet its accuracy relies heavily on sonographers’ skill proficiency. Sonographers must skillfully manipulate probes to obtain standardized sectional images, develop eye-hand coordination, and mentally reconstruct 2D images into 3D—a skill hard to master [1]. Therefore, training is essential for learners. International Society of Ultrasound in Obstetrics and Gynecology (ISUOG) recommended a minimum of 100 hours of supervised scanning, including at least 100 obstetric scans [2]. However, traditional training methods relying on practice with pregnant women present significant limitations. Prolonged supine position can easily lead to discomfort. Training increases unnecessary exposure of the fetus to ultrasound bioeffects. Additionally, traditional training follows a “see one, do one, teach one” approach [3], offering learners limited exposure to abnormalities. Trainees are dissatisfied with existing training due to limited time to practice on ultrasound machines [4].

Ultrasound simulators provide additional practicing opportunities. High fidelity ultrasound simulators enable repeated practice in a safe, controlled, and stress-free learning environment, and have been shown to improve the accuracy of fetal biometry measurements, reduce examination time, and enhance sectional image quality [5–8].

However, gaps persist in current obstetric ultrasound simulation training. First, existing trials focus narrowly on isolated tasks (e.g., fetal biometry, head anatomy) [3,9–11], lacking comprehensive curricula. Mid-trimester obstetric ultrasound—complex and time-intensive—remains rarely integrated into traditional residency programs [4], necessitating a structured, comprehensive course for its inclusion. Second, few multi-center studies validate skill improvement from such training [9], despite urgent demand for time-efficient programs in busy residencies. Third, little research explores simulation benefits across trainee experience levels: while consensus supports early-stage simulation [12], concerns about “expertise reversal effect” remain unaddressed [9], leaving a gap in training design for mixed-experience cohorts.

Thus, we designed a 4-hour modular simulation training course to enable residents to conduct detailed mid-trimester obstetric ultrasound scans. We hypothesized that deliberate practice in this short-term training would improve residents’ skills, thereby facilitating their transition to subsequent clinical practice. This study evaluated the course’s effectiveness among both inexperienced and experienced residents via two Kirkpatrick levels: measuring skill improvements within a simulated environment and assessing course satisfaction [13].

## Materials and methods

### Study design

**Fig 1:**
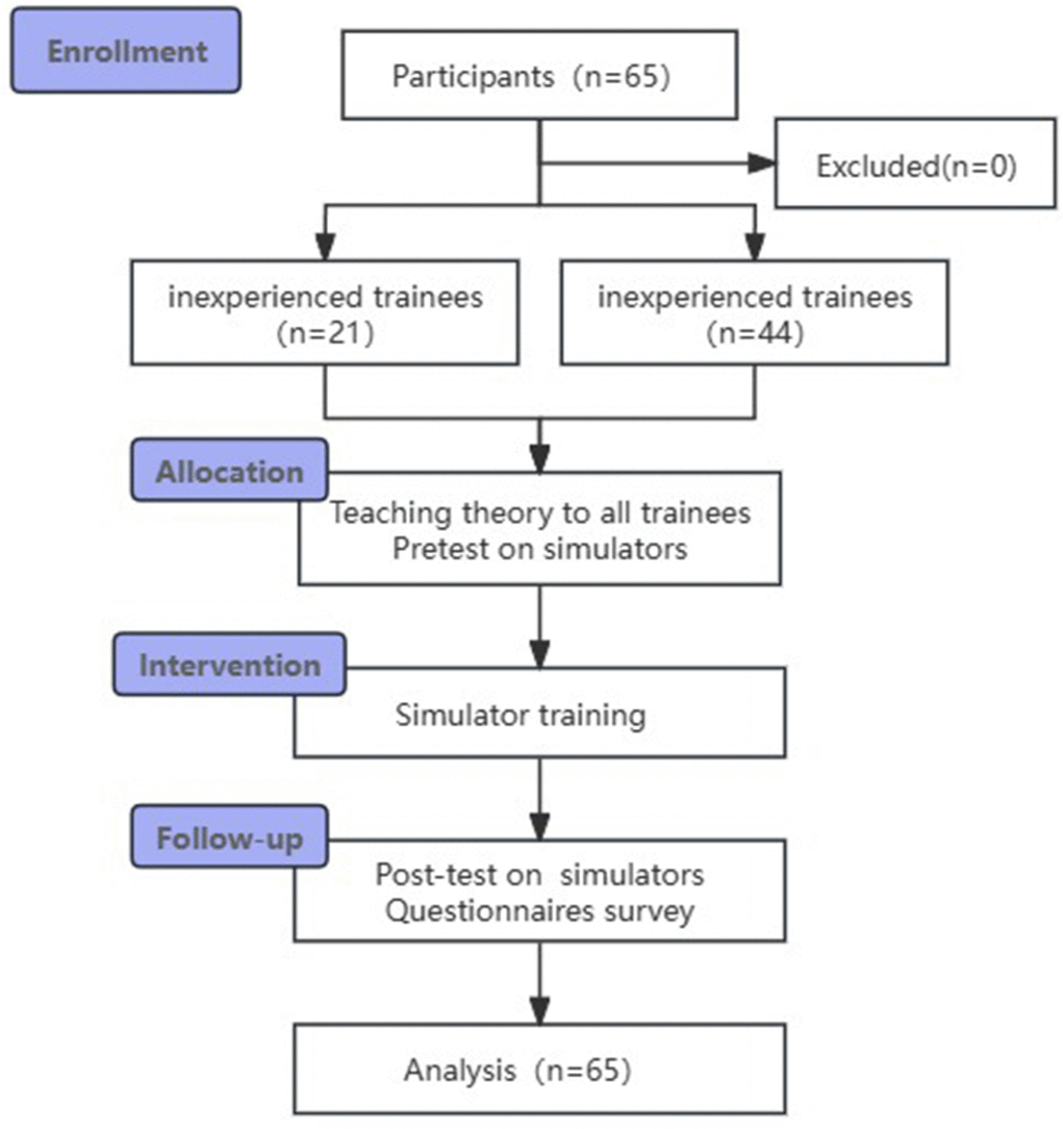
Flowchart of the study.

This prospective study used a pre-test–post-test design (Fig 1). After enrollment, participants first received theoretical instruction and completed a pre-test to establish baseline competency, then participated in structured simulation training followed by a post-test to quantify skill acquisition, and finally completed a standardized questionnaire assessing training perceptions and experiences. The study was approved by the Ethics Committee of the Third Xiangya Hospital of Central South University (No. 2020-S375). All participants provided written informed consent, and all procedures adhered to the Helsinki Declaration.

### Participants

Participants were selected in two steps: First, eligible residents were identified from the three centers (The Third Xiangya Hospital of Central South University, Hunan Provincial Maternal and Child Health Care Hospital, Nanjing Drum Tower Hospital) using predefined inclusion/exclusion criteria. Inclusion criteria: (1) first year residents without any obstetric ultrasound training experience (inexperienced trainees); (2) residents who had accrued more than one year of hands-on clinical training (experienced trainees). Exclusion criteria were: (1) prior obstetric ultrasound simulation training experience; (2) being unable to commit to the full 4-hour modular training and pre/post-test assessments. Second, all eligible residents were invited to participate voluntarily.

Sample size was determined by trainee availability. A total of 65 volunteer residents were enrolled between September 2020 and December 2021. These trainees, along with 24 certified obstetric ultrasound experts, previously participated in a skill assessment to collect validity evidence for the Obstetric Ultrasound Competency Assessment Tool (OUCAT) [14]; expert assessment data were used to establish pass/fail and mastery learning levels.

### Simulation training course

The course is designed to be introduced prior to trainees entering clinical practice or implemented as refresher training. To ensure standardized delivery across centers, training content was uniformly defined based on ISUOG guidelines [15,16]. A detailed training list (Table 1 and S1 Table) was developed, outlining 123 core tasks (covering preparation, fetal biometry/wellbeing assessment, anatomical survey, etc.) that all trainees must master—these tasks were identical for every participant, regardless of their experience level or participating center.

**Table 1:** Modules of the short-term simulation-based ultrasound training course.

Modules	Training contents
**Module 1**	Preparations, general information, examination on head and face.
**Module 2**	Examination on fetal heart.
**Module 3**	Examination on neck, chest, and abdomen.
**Module 4**	Examination on spine, limbs, placenta, umbilical cord, amniotic fluid, maternal uterine/adnexa. Conclusion.

The course structure followed a fixed modular design, with no deviations allowed. Given the complexity of mid-trimester obstetric ultrasound examinations and trainees’ difficulty mastering the complete procedure in one session, this course employs a modular design inspired by McConnaughey’s focused cardiac ultrasound training [17]. The organization of 4 task modules was informed by the need for systematic scanning, adherence to conventional sequences in guidelines, and the practicality of skill training. This modular approach allows trainees to engage with manageable tasks iteratively during each training session.

### Training

Prior to simulation training, all participants completed standardized didactic modules consisting of PowerPoint-delivered lectures detailing fundamental concepts and clinical performance standards for mid-trimester fetal ultrasound examinations. Lecture content was identical across centers (uniformly distributed PPT, 1-hour duration). All instructors also completed a 30-minute online training to ensure consistent key concept explanations.

**Fig 2:**
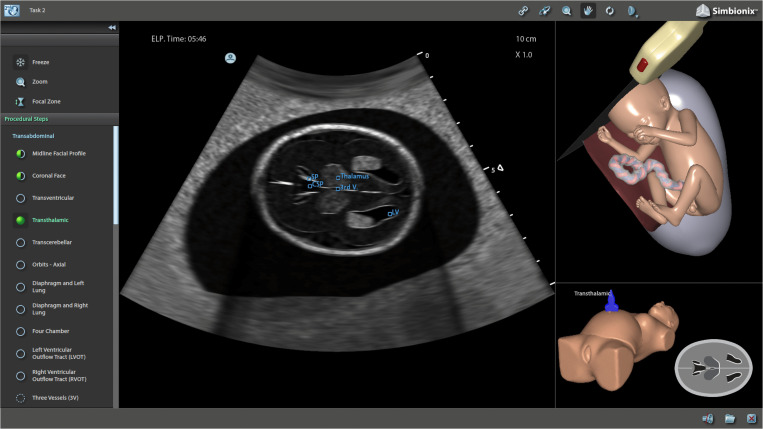
Simulator interface of the obstetrical ultrasound training module. The virtual fetus’s transthalamic plane was displayed center-screen, clearly showing anatomical structures like the septum pellucidum, thalamus, third ventricle, and lateral ventricle. The upper right corner depicted the ultrasound probe’s 3D orientation relative to the virtual fetus; concurrently, a schematic of the standardized transthalamic plane appeared in the lower right. When the probe was maneuvered to the prescribed plane, its color shifted to dark blue, signifying accurate positioning.

All centers used the same U/S Mentor simulator (Simbionix, Israel) for skill training, with uniform software versions and identical virtual patient cases (a 20-week-gestation fetus with normal anatomy) to eliminate variability from simulator settings or case complexity. Trainees generated real-time virtual ultrasound images by maneuvering the probe on the simulated abdomen, with simultaneous 3D anatomical guidance and probe orientation feedback (Fig 2).

Trainees used a dynamic self-scheduling system to select practice slots via a shared online document. They practiced in small groups of 3–4, each supervised by a dedicated instructor, following a standardized protocol: instructors first demonstrated procedural steps, then trainees took 30-minute turns (to prevent fatigue) for repeated practice—1 hour per module (4 modules total, 4 hours overall). Instructors provided on-demand assistance and feedback throughout. A standardized checklist (S1 table) was used to guarantee that trainees obtained guidance covering all core tasks. No concurrent training with real pregnant patients was allowed during simulation.

### Assessment

Trainees completed all task modules within one week after the pre-test, with the post-test administered immediately following the intervention. Assessments were conducted using simulators with a normal fetal module, as the evaluation focused on basic operational skills. Trainees were asked to perform mid-trimester obstetric ultrasound on simulators within 30 minutes during assessment. Evaluation videos were distributed to two consultants for scoring, with average score used as final score. Each video was sequentially numbered, with both participants’ personal information and their pre-test/post-test statuses blinded to assessors. OUCAT [14], a validated checklist, was administered to quantify participants’ skill levels. Pass/fail score was established by using contrast group method. A cutoff score was set to minimize the number of passing inexperienced trainees and falling experts in previous study [14,18]. Mastery learning levels, for the overall examination and each task module, were defined as average scores achieved by experts in counterparts [14,18]. The average proficiency level of qualified obstetric ultrasound experts represents the recognized competency for mid-trimester obstetric ultrasound. Based on previous research results, 45 points was taken as the pass/fail score, and mastery learning level was established at 70 points [14].

### Questionnaire survey

Researchers used Likert scales (1–5 points) before and after training to learn about participants’ interest and confidence in conducting obstetric ultrasound examinations [19]. After training, participants completed an anonymous questionnaire (Table 2). Researchers used the 1–5 points Likert scale to know about trainees’ views about operability (Q1-Q3) and fidelity (Q4-Q6) of the simulator, simulation training effectiveness (Q7-Q9), and overall comments on the program (Q10-Q12). The content of the questionnaire was developed with reference to previous questionnaires targeting simulators [20] and upon consultation with obstetric ultrasound experts. Trainees’ feedback was summarized into three aspects: Con (strongly disagree or disagree, 1–2 points), Neutral (uncertain, 3 points), and Pro (agree or strongly agree, 4–5 points).

**Table 2:** Questionnaire survey of the simulator and the simulation training program.

Questions	Pro	Neutral	Con	Mean	SD
Q1. I can smoothly optimize images and perform measurements as well as conducting other operations on the simulator.	54	4	2	4.23	0.79
Q2. Operating the simulator is the same as operating a real ultrasound equipment.	36	18	6	3.53	0.72
Q3. I can smoothly operate the simulator for second trimester obstetric ultrasound examinations.	49	9	2	4.00	0.71
Q4. The simulator shows clear images.	43	6	1	4.17	0.67
Q5. The landmark anatomical structures are clearly displayed on the simulator.	49	11	0	4.13	0.70
Q6. The simulator shows the same images as those of a real ultrasound equipment.	40	15	5	3.60	0.67
Q7. The simulator can improve my ability to obtain standardized obstetric ultrasound planes.	58	2	0	4.37	0.55
Q8. The simulator can help me form organized thinking of examination.	59	1	0	4.35	0.52
Q9. The simulator can improve my ability to examine pregnant women in clinical work.	54	5	1	4.23	0.67
Q10. Simulation skill training can replace traditional training on pregnant women.	27	18	15	3.33	1.00
Q11. My overall comments on the simulator (1–5 points, with higher scores indicating more support)	45	13	0	4.18	0.77
Q12. I will recommend simulation-based obstetric ultrasound examination training to others.	49	10	1	4.12	0.74

### Statistical analysis

SPSS 22.0 statistical software was used for statistical analysis. Comparison of measurement data following normal distribution between groups was conducted through t-test, and comparison before and after training was conducted by paired t-test. Non-normally distributed measurement data were compared by Wilcoxon signed-rank test. McNemar’s test was used to compare passing rate of trainees before and after training. Cronbach’s α was calculated to evaluate the reliability of items in the questionnaire survey. P < 0.05 was considered to be statistically significant.

## Results

### Demographics

Participants included 2 males and 63 females. Inexperienced trainees had a significantly lower median age (25 years, IQR 23–27) than experienced trainees (29 years, IQR 26–34; P < 0.001). Educational backgrounds: inexperienced trainees (15 bachelor’s, 6 master’s); experienced trainees (34 bachelor’s, 9 master’s, 1 PhD). No statistically significant intergroup difference in education was noted (P = 0.623).

### Outcome of assessment

**Fig 3:**
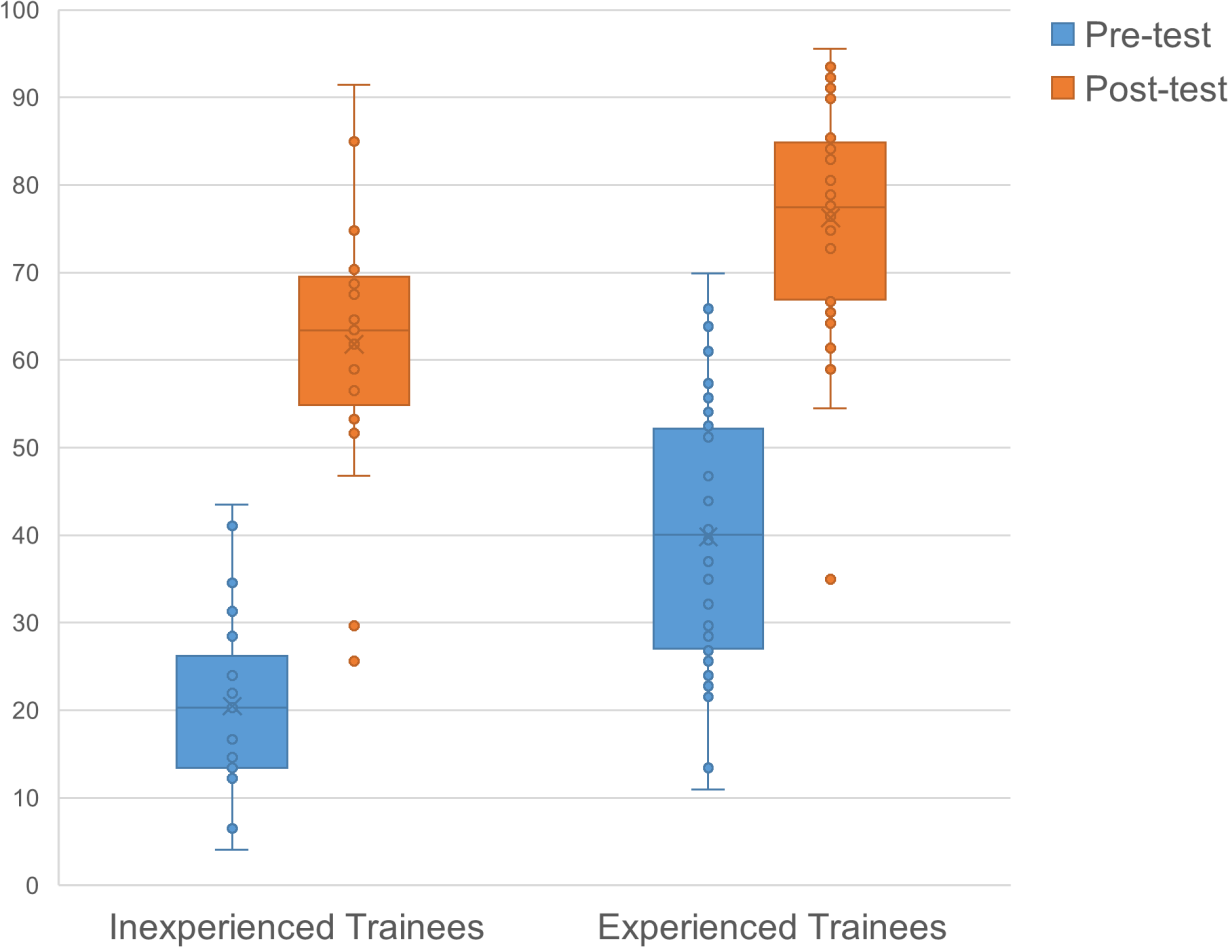
OUCAT scores of trainees in the pre-test and post-test.

Simulation training significantly improved trainees’ operational skills. OUCAT scores increased from 20.48 ± 10.64 to 61.80 ± 15.40 for inexperienced trainees (P < 0.001), and from 39.83 ± 15.00 to 76.26 ± 12.16 for experienced trainees (P < 0.001) (Fig 3). Performance improvement did not differ between groups (41.33 ± 15.47 for inexperienced trainees vs. 36.43 ± 14.32 for experienced trainees, P = 0.213).

The passing rate increased from 0% to 90.5% for inexperienced trainees, and from 31.8% to 97.7% for experienced trainees (all P < 0.001). The mastery learning rate increased from 0% to 23.8% for inexperienced trainees, and from 0% to 70.5% for experienced trainees (all P < 0.001).

Skill improvement varied by module and group. Table 3 presents the scores and mastery learning rates for each task module. Pre-test revealed significantly lower scores among trainees compared to experts across all modules (all P < 0.001). In post-test, Inexperienced trainees demonstrated comparable performance to experts in modules 1 (P = 0.535), 2 (P = 0.320), and 3 (P = 0.247), but remained inferior in module 4 (P = 0.003). Experienced trainees showed no significant differences from experts in modules 1 (P = 0.382), 2 (P = 0.107), and 4 (P = 0.094), but outperformed them in module 3 (P = 0.002). Mastery learning rates post-training varied. Inexperienced trainees attained the highest rate in module 1 (57.1%), with rates ≤33% in remaining modules. Experienced trainees achieved peak mastery in module 3 (75.0%).

**Table 3:** OUCAT score and mastery learning level of trainees in each task module before and after training.

		OUCAT scores						Marstery learning level			
	Task modules	Inexperienced trainees		Experienced trainees		Experts		Inexperienced trainees		Experienced trainees	
		Mean	SD	Mean	SD	Mean	SD	Pass/fail	Marstery learning Rate	Pass/fail	Marstery learning Rate
Pre-test	Task module 1	9.31	4.92	14.73	4.67	22.15	3.74	0/21	0%	2/42	4.5%
	Task module 2	2.56	2.53	6.65	5.20	16.67	4.35	0/21	0%	3/41	6.8%
	Task module 3	3.50	2.61	8.63	3.21	13.11	2.46	0/21	0%	2/42	4.5%
	Task module 4	5.11	3.50	9.36	5.26	17.80	3.62	0/21	0%	3/41	6.8%
	Total	20.48	10.64	39.83	15.00	70.33	10.67	0/21	0%	0/44	0%
Post-test	Task module 1	21.37	4.66	22.98	3.65	–	–	12/9	57.1%	29/15	65.9%
	Task module 2	14.83	5.42	18.17	4.63	–	–	7/14	33.3%	29/15	65.9%
	Task module 3	12.08	3.42	15.14	2.52	–	–	7/14	33.3%	33/11	75.0%
	Task module 4	13.51	5.45	19.63	4.44	–	–	5/16	23.8%	30/14	68.2%
	Total	61.80	15.40	76.27	12.16	–	–	5/16	23.8%	31/13	70.5%

### Questionnaire survey

65 questionnaires were distributed and 60 were recovered, with a questionnaire recovery rate of 92.3%. Cronbach’s α coefficient for items in the questionnaire was 0.891, A Cronbach’s α coefficient > 0.7 indicates good internal consistency reliability [21]. Participants showed a high degree of interest in learning obstetric ultrasound before and after training (4.25 ± 0.69 vs. 4.28 ± 0.49, P = 0.982). After training, participants’ confidence in completing obstetric ultrasound examination was significantly improved (2.66 ± 1.12 vs. 3.98 ± 0.50, P < 0.001).

Overall, trainees were satisfied with the obstetric ultrasound simulation training course (Table 2). Average scores of all questions were higher than the median value of 3 points, among which the highest 3 scoring metrics were about simulation training effectiveness (Q7-Q9). The main disagreements were focused on the image style and the operation interface of the simulator (Q2, Q6). A considerable number of trainees disagree that simulation training can supplant traditional training (Q10).

## Discussion

This study developed a novel obstetric ultrasound simulation training course. Through short-term deliberate practice, participants showed significant operational skill improvement. While simulation offers a stress-free setting and additional practice opportunities, the traditional belief that “the patient is the best teacher” remains prevalent, further evidence is needed to fully establish the effectiveness of obstetric ultrasound simulation [22]. Burden et al. found that trainees with little experience achieved statistically significant improvements in biometric measurement accuracy and scanning efficiency following focused simulator training, with final performance approaching certified experts [6]. Rosen showed that 45 minutes of fetal brain anatomy survey training on simulators improved all trainees’ image quality, anatomical landmarks identification, and measurements [11]. Notably, simulation-induced skill gains may translate to better clinical outcomes: Andreasen reported simulation-trained obstetricians had a 31.9% higher fetal weight estimation accuracy and improved image quality when examining real pregnant patients [9]. Our findings align with previous research [6,9,23,24], supporting simulation-based training’s value in obstetric ultrasound. However, the course’s impact on skill improvement still requires validation through a randomized controlled trial.

Our study observed performance improvement among both inexperienced and experienced residents. Prevailing consensus supports introducing simulation training during the initial phase of education [1,12]. The learning curve was non-linear, and the improvement in early stages was often more significant. Introducing simulation training in early stages of learning could help improve the minimum skill level of learners [24]. Besides, Andreasen mentioned that based on the expertise reversal effect theory, providing simulation training for experienced obstetricians may change their habits and have negative impacts [9]. However, this study found that simulation training could also benefit experienced trainees. A possible explanation would be that experienced trainees included were not skilled experts. This aligns with Andreasen et al, who observed that offer simulation training to experienced obstetrician also improved their diagnostic accuracy [9]. In aviation training, most airlines conduct regular recurrent training using flight simulators to maintain safety standards [3]. Introducing periodic short-term simulation training for experienced trainees may also help standardize their operation and maintain skill proficiency.

While over 90% of trainees met the passing standard, only 23.8% of inexperienced trainees and 70.5% of experienced ones reached the mastery learning level. Considering patient safety, simulation training often needs to elevate learners to the mastery learning level [25]. Analysis of module-specific mastery learning rates showed variable trainee responses: inexperienced trainees had the highest mastery learning rate in Module 1, with gradual declines in Modules 2–4, differing from experienced trainees’ learning trajectory. Mastery learning theory holds that trainees should master current content before progressing to new material [26]. However, the training time of each task module in the course was fixed and carried out in sequence. We speculate trainees who failed to master earlier modules still advanced to new content—posing greater challenges for inexperienced trainees with low baselines and leading to poor grasp of subsequent modules. Thus, integrating mastery learning principles into the simulation course, as McGaghie suggested [27], is recommended.

Trainees’ satisfaction reflects Level 1 (Reaction) in the Kirkpatrick model [13]. Positive experiences boost engagement and training effectiveness. Our survey found most trainees favored simulation training and were willing to accept it, with the highest scores for the training’s effectiveness. Trainees saw it as beneficial for obtaining standardized ultrasound planes, forming organized examination thinking and potentially improving their ability to examine real pregnant women. However, a minor drawback was that the simulator’s operational interface and imaging style negatively impacted some participants’ learning experience, as noted in Ostergaard’s survey [20]. Moreover, most trainees recognized simulation training as only a valuable adjunct to traditional clinical training, which offers more intricate real-life scenarios and knowledge than simulations. Our survey found simulation training significantly boosts learners’ confidence in obstetric ultrasound examinations—this confidence comes from improved technical proficiency, as Tolsgaard showed confidence ties closely to learners’ technical skills and image analysis abilities. [28].

Our simulation training program incorporates deliberate practice principles, with trainees pursuing clear objectives through intensive structured repetition across four modules. It offers dual feedback: real-time simulator data (3D anatomical visualization, probe position visualization) plus expert guidance via live demonstrations and corrective adjustments—enabling efficient skill acquisition. As a key element of simulation-based medical education [27], deliberate practice was successfully used in McConnaughey’s Focused Cardiac Ultrasound training, yielding faster skill acquisition than traditional curriculum [17].

Our findings offer implications for optimizing obstetric ultrasound residency training. For inexperienced trainees, the 4-hour modular course provides a feasible pre-clinical skill foundation. Traditional training often places novices in real patient encounters without prior procedural familiarity, leading to longer learning curves and potential ethical concerns [25,29]. Our data shows this simulation course raises their OUCAT scores and boosts passing rate to 90.5%, enabling entry into clinical practice with basic probe skills and standardized scanning thinking. Institutions could integrate it as a mandatory pre-rotation module to ensure core task mastery before real scans. For experienced trainees, the course addresses a gap in traditional training: operational standardization. Though experienced trainees had higher baseline scores, their 70.5% post-training mastery rate confirms the course’s value in correcting “informal habits” formed during unstructured clinical practice. For busy departments, this 4-hour refresher training course is more time-efficient than extended workshops and better suited to residents’ heavy workloads. Furthermore, after such supervised short-term training, trainees can proceed to self-directed learning. This not only facilitates skill retention but also reduces supervisors’ teaching burden [17,30].

Several limitations should be acknowledged: 1. The pre-test-post-test design lacked a control group, potentially affecting the validity of conclusions. 2. The extent to which skill improvement transfer to clinical behavior changes, as well as long-term retention, remains unknown. 3. Variability in simulator types may influence training outcomes. 4. The small sample size (due to limited resident availability) restricts generalizability. 5. The course focused on basic operational skills and excluded abnormal obstetric scenarios. 6. Class hours were allocated based on practicality rather than systematic difficulty assessment.

## Conclusions

The short-term simulation-based obstetric ultrasound training course was associated with improved skills. Trainees also reported high level of course satisfaction, aligning with Kirkpatrick Level 1 (Reaction) and Level 2 (Learning) standards. Notably, the 4-hour duration is relatively short, and findings are limited to skill changes within simulated environment, not real clinical practice. Given these limitations, the course’s main value is as a foundational training adjunct: bridging theory and initial clinical practice for inexperienced trainees, and supporting operational standardization for experienced ones. Future research should integrate mastery learning principles to address low mastery rates and validate clinical skill transfer via randomized controlled designs with longer follow-up.

## Supporting information

S1 Table Simulation-based obstetric ultrasound training course. (PDF)
