# Supplementary material for: Short-term simulation training course improves basic obstetric ultrasound operational skills of residents: A multi-center study
Source: PLoS One. 2025 Dec 26;20(12):e0328122. doi: 10.1371/journal.pone.0328122 (PMC12742739; doi:10.1371/journal.pone.0328122)
Supplement: S1 Table — (PDF) [file pone.0328122.s001.pdf]

S1 Table. Simulation-based obstetric ultrasound training course

Modules	Sections		Training contents
Task module 1	Preparations	1.1	Check the name of the pregnant woman
		1.2	Check the age of the pregnant woman
		1.3	Check the last menstruation date and the week of gestation
		1.4	Check the history of pregnant and birth, genetic diseases, and surgery
		1.5	Inform the pregnant women about the aims, contents, and limitations of obstetric ultrasound examination
	General information	1.6	Check the fetal number
		1.7	Check the fetal presentation
		1.8	Measure fetal heart rate
		1.9	Check fetal movement
	Head	1.10	Check the skull bone
		1.11	Obtain the transventricular plane
		1.12	Check the midline falx on the transventricular plane
		1.13	Check the lateral ventricles on the transventricular plane
		1.14	Check the choroid plexus on the transventricular plane
		1.15	Measure the width of the lateral ventricle
		1.16	Obtain the transthalamic plane
		1.17	Measure biparietal diameter (BPD)
		1.18	Measure head circumference (HC)
		1.19	Check the cavum septi pellucidi on the transthalamic plane
		1.20	Check the thalami on the transthalamic plane
		1.21	Check the third ventricle on the transthalamic plane
		1.22	Check the insula on the transthalamic plane
		1.23	Obtain the transcerebellar plane
		1.24	Check the cerebellar hemispheres
		1.25	Check the cerebellar vermis
		1.26	Measure the transverse cerebellar diameter
		1.27	Check the cisterna magna
		1.28	Measure the width of cisterna magna
	Face	1.29	Obtain the axial view of the orbits
		1.30	Check the orbits
		1.31	Check the lenses
		1.32	Obtain the midline facial profile
		1.33	Check the nasal bone
		1.34	Obtain the coronal plane of the face
		1.35	Check the nose
		1.36	Check the lips

Task module 2	Heart	2.1	Obtain the four-chamber view of the heart
		2.2	Check the location of the heart
		2.3	Check the orientation of the cardiac apex
		2.4	Check the cardiac axis
		2.5	Check the heart/chest ratio
		2.6	Check the inferior vena cava (IVC) and the connection of IVC to right atrium
		2.7	Check the right atrium
		2.8	Check the left atrium
		2.9	Check the pulmonary veins and the connection of pulmonary veins to left atrium
		2.10	Check the interatrial septum
		2.11	Check the foramen ovale
		2.12	Check the left ventricle
		2.13	Check the right ventricle
		2.14	Check the interventricular septum
		2.15	Check the mitral valve
		2.16	Check the tricuspid valve
		2.17	Evaluate blood flow signals in the four-chamber view by CDFI
		2.18	Obtain the left ventricular outflow tract plane of the heart
		2.19	Check the left ventricular outflow tract
		2.20	Check the ascending aorta
		2.21	Evaluate blood flow signals in the LV outflow tract by CDFI
		2.22	Obtain the right ventricular outflow tract plane of the heart
		2.23	Check the right ventricular outflow tract
		2.24	Check the pulmonary artery
		2.25	Evaluate blood flow signals in the right ventricular outflow tract by CDFI
		2.26	Obtain the three-vessel view of the heart
		2.27	Obtain the three-vessel and trachea view of the heart
		2.28	Check the ductal arch
		2.29	Check the aortic arch
		2.30	Check the superior vena cava
		2.31	Check the trachea
		2.32	Evaluate blood flow signals in the three-vessel and trachea view by CDFI
Task module 3	Neck	3.1	Check the presence of masses in the neck
		3.2	Check the presence of skin edema in the neck
		3.3	Check the presence of umbilical cord around the neck
	Chest	3.4	Continuously check the chest on the axial view

		3.5	Obtain the sagittal view of the diaphragm and left lung
		3.6	Obtain the sagittal view of the diaphragm and right lung
		3.7	Obtain the coronal view of the diaphragm
		3.8	Check the shape of the chest
		3.9	Check the left lung and right lung
		3.10	Check the diaphragm
	Abdomen	3.11	Obtain abdominal circumference (AC) plane
		3.12	Measure AC
		3.13	Check the stomach bubble
		3.14	Check the liver
		3.15	Check the gall bladder
		3.16	Obtain axial view of kidneys
		3.17	Obtain coronal view of kidneys
		3.18	Obtain sagittal views of kidneys
		3.19	Obtain umbilical cord insertion plane
		3.20	Check the abdominal wall
		3.21	Check the intestinal tract
		3.22	Obtain the axial view of the bladder and umbilical arteries, with CDFI
		3.23	Check the bladder
		3.24	Check the umbilical arteries
Task module 4	Spine	4.1	Obtain the midsagittal view of spine
		4.2	Continuously check the spine on axial view
		4.3	Obtain the coronal view of the spine
		4.4	Check the continuity of the spine
		4.5	Check the position of conus medullaris
	Limbs	4.6	Check the left humerus
		4.7	Measure humerus length (HL)
		4.8	Check the left ulna and radius
		4.9	Check the left hand
		4.10	Check the right humerus
		4.11	Check the right ulna and radius
		4.12	Check the right hand
		4.13	Check the left femur
		4.14	Measure femur length (FL)
		4.15	Check the left tibia and fibula
		4.16	Check the left foot
		4.17	Check the right femur
		4.18	Check the right tibia and fibula
		4.19	Check the right foot
	Placenta，	4.20	Check the umbilical cord insertion site into the placenta

	umbilical cord and amniotic fluid	4.21	Continuously check the placenta
		4.22	Measure the thickness of the placenta
		4.23	Measure the distance between the placenta and internal uterine cervix orifice
		4.24	Check the umbilical cord placental insertion site and abdominal wall insertion site, check the number of umbilical vessels
		4.25	Measure amniotic fluid index
	Maternal uterus/adnexa	4.26	Obtain the midsagittal plane of the cervix
		4.27	Check the uterine cervix
		4.28	Measure cervical length on the midsagittal plane of the cervix
		4.29	Check the maternal uterus
		4.30	Check the adnexa of the maternal uterus
	Conclusions	4.31	Summarize the examination
